# Multi-Omics Analysis Reveals Chronic Cisplatin Exposure Is Associated with Metabolic Rewiring Toward Glutathione Metabolism to Support Redox Adaptation in High-Grade Serous Ovarian Cancer

**DOI:** 10.3390/cancers18121945

**Published:** 2026-06-15

**Authors:** Ashlyn Conant, Kayla Sanchez, Shreya Patil, Ethan Nyein, Tise Suzuki, Gary Yu, Marlon Maus, Salvador Soriano, Christian Hurtz, Juli J. Unternaehrer

**Affiliations:** 1 Department of Basic Sciences, School of Medicine, Loma Linda University, 11085 Campus Street Mortensen Hall 219, Loma Linda, CA 92354, USA; abartlett@students.llu.edu (A.C.); suzukit@southern.edu (T.S.); gyu@llu.edu (G.Y.); ssoriano@llu.edu (S.S.); 2 The Taub Institute for Research on Alzheimer’s Disease and the Aging Brain, Vagelos College of Physicians and Surgeons, Columbia University, New York, NY 10032, USA; ks4561@cumc.columbia.edu; 3 Division of Cancer Sciences, Department of Basic Sciences, Loma Linda University, Loma Linda, CA 92354, USA; shreyapatil@llu.edu (S.P.); churtz@llu.edu (C.H.); 4 Department of Gynecology and Obstetrics, Loma Linda University, Loma Linda, CA 92354, USA; 5 School of Public Health, The University of California, Berkeley, CA 94720, USA; maus1@berkeley.edu

**Keywords:** ovarian cancer, therapy resistance, metabolism, glutathione, taurine, beta oxidation, ROS buffering, redox-maintenance

## Abstract

Background: Platinum-based chemotherapy is the frontline treatment for high-grade serous ovarian cancer (HGSOC); however, the development of therapy resistance greatly limits clinical response. Increasing evidence suggests that platinum agent-driven metabolic programming, particularly within redox-associated pathways, may contribute to chemoresistance. Methods: A syngeneic pair of patient-derived HGSOC cell lines representing cisplatin-sensitive (SE) and cisplatin-resistant (CR) states were evaluated using a multi-omics approach. Differential metabolite abundance and gene expression were assessed, followed by gene set and pathway enrichment analyses to identify coordinated metabolic shifts. In silico analysis of an additional sensitive and resistant HGSOC cell line validated the glutathione pathway upregulation seen in the patient-derived model. The functional contribution of the glutathione pathway on cisplatin resistance was evaluated following glutathione inhibition. Results: Chronic cisplatin exposure induced extensive metabolic rewiring in CR cells, characterized by enrichment of glutathione metabolism at both the metabolite and gene levels. Increased reduced glutathione was observed alongside upregulation of key enzymes involved in its de novo biosynthesis, recycling, and utilization, consistent with enhanced detoxification capacity relating to cisplatin-induced oxidative stress. Additionally, taurine was highly enriched, further highlighting a metabolic shift towards enhanced antioxidant mechanisms. CR cells also demonstrated an increase in NADPH-generating pathways, including amino acid metabolism and fatty acid β oxidation, to support redox balance and biosynthetic demands of increased glutathione metabolism. Transcriptional remodeling of the γ-glutamyl cycle further indicated a shift toward increased glutathione turnover, suggesting that the coordinated changes seen may define a metabolic state enhanced in oxidative stress tolerance and therapeutic resistance. These transcriptional changes were also seen in another model of platinum sensitivity/resistance, indicating a conserved response associated with platinum-induced resistance. Finally, concurrent cisplatin treatment and glutathione inhibition significantly increased sensitivity within the CR cells. Conclusions: These findings suggest that cisplatin-resistant cells, previously exposed to a platinum-based agent, may undergo distinct metabolic rewiring towards antioxidant pathways to survive chronic chemotherapeutic stress. Targeting components of these systems may represent a viable strategy to overcome platinum resistance and improve therapeutic outcomes.

## Simple Summary

Increasing evidence suggests that platinum-driven metabolic programming, particularly within redox-associated pathways, may contribute to the development of chemoresistance in high-grade serous ovarian cancer (HGSOC). A multi-omics approach was utilized to assess the metabolomic changes on the metabolite and gene levels, using a pair of cisplatin-sensitive and -resistant patient-derived HGSOC cells. Chronic cisplatin exposure induced distinct metabolic rewiring in chemoresistant cells, defined by enrichment of glutathione metabolism. Increased reduced glutathione, as well as enzymes involved in its de novo biosynthesis, recycling, and utilization, were upregulated, consistent with enhanced detoxification capacity. The resistant cells also demonstrated enrichment of enzymes and substrates utilized in NADPH-generating pathways, which may support redox balance and biosynthetic demands of increased glutathione metabolism. Inhibition of glutathione production increased cisplatin sensitivity, indicating that glutathione contributes functionally to the resistant phenotype. These findings were validated in an additional sensitive and resistant cell line and further suggest that the coordinated changes associated with chronic cisplatin exposure may define a therapeutically vulnerable metabolic state: enhanced oxidative stress tolerance and therapeutic resistance.

## 1. Introduction

High-grade serous ovarian cancer (HGSOC) is among the most lethal gynecologic malignancies and is therefore managed using an aggressive multimodal treatment approach [1]. Maximal cytoreductive surgery is a critical first step in initial management to allow for both accurate FIGO (federation of gynecology and obstetrics) staging and the achievement of minimal (to no) visible residual disease (MRD) [2]. Following surgical debulking, patients typically receive platinum–taxane chemotherapy, administered in multiple cycles across four to six months [3,4]. Most patients respond well to this regimen; however, 70–90% of patients experience recurrence. Recurrent tumors are treated with additional rounds of platinum-based therapy and, in many cases, demonstrate an increase in platinum resistance following recurrence [5]. Thus, recurrence and resistance represent a major barrier and area of treatment failure in the field [6,7]. As tumor cells in HGSOC are exposed to frequent, repeated, and prolonged platinum treatment, it is essential to understand how chronic exposure shapes tumor cell biology and contributes to the development of therapy resistance.

The mechanisms driving therapy resistance in ovarian cancer are complex and multifactorial, involving adaptive responses that are shaped by cell type, as well as alterations in DNA damage repair (DDR) pathways, genomic instability, epigenetic remodeling, and metabolic reprogramming [8]. The Warburg effect, or increased aerobic glycolysis, is one of the most well studied metabolic shifts that occurs in cancer and is exploited therapeutically in some settings [9]. Beyond this metabolic shift, cancer cells frequently undergo complex, global metabolic rewiring following treatment to support biosynthetic requirements, maintain redox balance, and mitigate cytotoxic damages [10,11]. Notably, these adaptations are not always uniform; distinct therapeutic agents impose unique selective pressures that drive drug-specific metabolic reprogramming, resulting in divergent survival strategies tailored to the mechanism of action of each treatment. This reprogramming enables tumor cells to sustain survival under cytotoxic stress by mobilizing and reallocating key resources, including ATP, amino acids, sugars, nucleotides, and other essential substrates. Most notably, metabolic plasticity enables tumor cells to increase the intracellular threshold for oxidative stress while repairing damage and undergoing phenotypic transitions that promote survival.

Cisplatin is a non-cell cycle-specific, alkylating therapeutic agent that intercalates into double-stranded DNA (both nuclear and mitochondrial) to create inter- and intra-strand crosslinks [12]. These crosslinks create significant structural distortion, inhibiting DNA transcription, repair, and synthesis, while halting cell cycle progression and promoting G2/M arrest (Figure S1). Consequently, DDR pathways are activated and enhanced to attempt repair before the cell undergoes induction of apoptosis. This drug is remarkably efficacious, as evidenced by a therapeutic paradigm for ovarian cancer that has remained largely unchanged for nearly three decades. However, chronic exposure to cisplatin may also induce many other cellular adaptations, beyond DDR alterations. Studies now suggest that sustained chemotherapy exposure can reshape metabolic and transcriptional pathways to support survival and growth [13,14]. Despite the growing interest in this area, the global metabolic consequences of prolonged cisplatin exposure in HGSOC remain understudied.

Multi-omics approaches have proven to be a powerful tool in characterizing transcriptional and metabolic alterations of cancer cells during therapeutic treatment [15]. By observing changes in metabolites and gene transcription, multi-omics profiling can reveal potentially targetable metabolic and transcriptional vulnerabilities that may not be completely apparent through genomic analyses alone. Understanding how tumor cell metabolism changes during repeated and chronic alkylating agent exposure may uncover novel mechanisms of resistance that might be exploited to improve treatment responses. Within the context of ovarian cancer, knowledge of metabolic adaptations following standard therapy may provide researchers and clinicians, alike, with information required to develop co-therapies that enable the standard platinum therapy to remain successful, thus avoiding resistance and recurrence [16].

In this study, we utilized a patient-derived, syngeneic model of HGSOC to determine the metabolic changes associated with chronic cisplatin treatment. By comparing metabolic and transcriptomic profiles of a chemo-naïve and chemoresistant pair of HGSOCs, we aimed to define trends in metabolic adaptations that accompany the development of chemoresistance. Defining these alterations may help better understand the effect of repeated and prolonged platinum-based agent exposure, contribute to the development of alternative recurrent treatment plans, and inform novel therapeutic innovation.

## 2. Materials and Methods

### 2.1. Collection and Generation of the Syngeneic Sensitive/Resistant Model

The collection, isolation, and use of the patient-derived cells used in this study were approved by the Loma Linda University (LLU) Institutional Review Board (IRB 58328). Following patient consent, tumor tissue was deidentified and collected by the Loma Linda University Cancer Center Biospecimen Laboratory (LLUCCBL) before transport to the laboratory for tissue processing. The patient sample (PDX4) was collected, preserved, and processed as previously described [17].

PDX4-sensitive (SE) and -resistant (CR) cells were generated as previously described [18].

### 2.2. Cell Culture

PDX4 SE and CR cells were cultured in a 3:1 mixture of HycloneTM Ham’s Nutrient Mixture F12 with L-glutamine (SH30026.01; Cytiva, Marlborough, MA, USA) and Dulbecco’s Modified Eagle’s Medium with high glucose (DMEM; 25-501), with supplementation of 5% FBS (Cell Culture Collective, Encinitas, CA, USA), 0.4 μg/mL hydrocortisone (H0888-1G; MilliporeSigma, Burlington, MA, USA), 5 μg/mL insulin (91077C-100MG; MilliporeSigma), 2 μg/mL isoprenaline hydrochloride (I5627-5G; MilliporeSigma), 24 μg/mL adenine (A8626; MilliporeSigma), 100 U penicillin, and 100 μg/mL streptomycin (25-512; Genesee Scientific, El Cajon, CA, USA) at 5% CO_2_. The cells were analyzed for mycoplasma contamination (PlasmoTestTM Mycoplasma Detection Kit (rep-pt1; InvivoGen, San Diego, CA, USA)) and their identity confirmed before and after establishment of resistance with Short Tandem Repeat (STR) validation (Laragen Inc., Culver City, CA, USA).

A2780 (sensitive) was a kind gift of Dr. Ann Klopp, MD Anderson. A2780 (cisplatin-resistant) was a kind gift from Dr. Dina Dinulescu (Harvard University). Cells were cultured in RPMI 20 1640 Medium with L-glutamine (SH30027.02, Cytiva) with supplementation of 10% FBS (Cell Culture Collective), 100 U penicillin, and 100 μg/mL streptomycin (25-512; Genesee Scientific) at 5% CO_2_.

### 2.3. Mass Spectrometry

Sample Preparation: A total of 1 × 10^6^ CR cells pre-treated with 12 μM cisplatin three days prior to collection. SE and CR cells were collected in 3 biologic replicates. Simple extraction and protein precipitation were completed by pelleting cells and adding 1 mL of 80% ice-cold methanol and incubating on ice for 5 min before vortexing. Cells were left at −20 °C for 30 min and pelleted at 15,000× *g* for 15 min at 4 °C. Collected supernatant was dried completely using SpeedVac (Thermo Fisher Scientific, Waltham, MA, USA) concentrator. Lysates were reconstituted in 100 uL of Acetonitrile:water(1:1 *v*/*v*) and transferred to the autosampler for analysis.

Mass Spectrometry: Bioactive compounds were separated using a 150 × 2.1 mm, 3 μm, 100 A Luna Omega Polar C18 Column (Phenomenex, USA) in both ESI positive and negative modes, employing a Thermo Scientific Vanquish UPLC system coupled with an Orbitrap Exploris 240 mass spectrometer (Thermo Fisher Scientific).

All separations were carried out with the column oven set to 40 °C. For ESI positive mode, the mobile phase A was 0.1% formic acid in water and 5 mM Ammonium Formate. The mobile phase B was 0.1% formic acid in IPA:Acetonitrile (90:10) and 5 mM Ammonium Formate. For ESI negative mode, the mobile phase A was 0.1% acetic acid in water and 5 mM Ammonium Acetate. The mobile phase B was 0.1% acetic acid in IPA:Acetonitrile (90:10) and 5 mM Ammonium Acetate. A 15 min gradient was employed for both positive and negative modes as follows: 0–5 min, 0–50% B; 5–6 min, 50–98% B; 6–10 min, 98% B; 10.1–15 min, 0% B at a flow rate of 0.3 mL/min. The injection volume was 10 µL for each analysis.

For positive ion mode, the spray voltage was adjusted at 3500 V, whereas for negative ion mode, it was adjusted at 2500 V. The flow rates of sheath gas and auxiliary gas were set at 50 and 10 arb, respectively. The ion transfer and vaporizer temperatures were controlled at 325 °C and 350 °C, respectively. The full scan resolution was 120,000 with a scan range from *m*/*z* 70 to 1000, while the ddMS2 resolution was set to 15,000. The dynamic exclusion duration was set to 3 s, with an intensity threshold of 5000.

Data Processing and Metabolite Identification: Raw mass spectrometry data was processed using Compound Discoverer software (Software Version 3.3; Thermo Scientific) for peak detection, retention time alignment, metabolite identification based on accurate precursor mass and MS2 fragmentation spectra to mzCloud, HMDB, ChemSpider databases within a mass tolerance of 5 ppm.

Statistical Analysis: Statistical analysis was conducted in R (version 4.5.0). Missing values were imputed by half-minimum value replacement, and the data was log_2_-transformed. Principal component analysis (PCA) was scaled with a Pareto scaling method to reduce the influence of highly abundant metabolites while preserving data structure. Samples were visualized in a two-dimensional score plot using top two variances explaining PCA components—PC1 and PC2 as reference axes. Differentially abundant metabolites between treatment and control groups were identified using an unpaired, two-tailed Student’s *t*-test. Metabolites were categorized as upregulated or downregulated based on the direction of log_2_ fold change and *p*-value relative to comparison group. Pathway enrichment analysis, overrepresentation, and string analyses was conducted in MetaboAnalyst 6.0 (http://www.metaboanalyst.ca/) using KEGG pathway library mapped to human metabolome and using Mitocarta3.0 (https://www.broadinstitute.org/mitocarta/mitocarta30-inventory-mammalian-mitochondrial-proteins-and-pathways, accessed on 23 April 2026).

### 2.4. Library Preparation and RNA Sequencing

Library preparation and RNA sequencing was completed and documented as previously described [18]. Differential gene expression analysis was performed using DESeq2, and *p*-values were adjusted for multiple hypothesis testing using the Benjamini–Hochberg procedure to control for false discovery rate (FDR). Genes with an adjusted *p*-value below 0.05 were considered significantly differentially expressed.

### 2.5. Pathway Analysis

Ingenuity Pathway Analysis (IPA; QIAGEN Inc., Hilden, Germany, https://digitalinsights.qiagen.com/IPA; accessed on 23 March 2026) was used for the identification of canonical/hallmark metabolomic pathways activated (|Z| > 2) among the DEGs obtained through the PDX4 RNA-seq analysis. Bar charts were created with GraphPad Prism v 10.5.0.

### 2.6. Gene Set Enrichment Analysis

Gene Set Enrichment Analysis was performed using GSEA Software Version v 4.4.0 (GSEA, www.broadinstitute.org/gsea, accessed on 27 October 2023). Expression datasets for PDX4 SE and PDX4 CR cells were analyzed against Hallmark_Reactive Oxygen Species Pathway and Kegg_ Glutathione Metabolism. The enrichment analysis used phenotype-based permutation with 1000 permutations. The chip platform “Human_Ensemble_Gene_ID_MSigDB.v2026.1.HS.chip” was used for mapping gene identifiers. Gene sets containing more than 10,000 genes and less than 5 genes were excluded. The enrichment statistic was set to “weighted,” and genes were ranked using the “log2_Ratio_of_Classes” metric. Gene lists were sorted in “real” mode and arranged in descending order.

### 2.7. Quantitative Real Time Polymerase Chain Reaction (RT-qPCR)

RNA from cells was isolated using the IBI Isolate DNA/RNA Reagent Kit (IB47602, IBI Scientific, Dubuque, IA, USA) according to the manufacturer’s recommendations. cDNA was synthesized from either 500 ng or 1 μg of total isolated RNA using Maxima First Strand cDNA Synthesis Kit (K1672; Thermo Fisher Scientific, Waltham, MA, USA). RT-qPCR was performed using Applied Biosystems^TM^ PowerUP^TM^ SYBR^TM^ Green Master Mix (A25778; Thermo Fisher Scientific) and gene primers (Actin FWD 5′-TGAAGTGTGACGTGGACATC-3′ REV 5′-GGAGGAGCAATGATCTTGAT-3′, GCLC FWD 5′-CAAGGACCGGCACAAGGA-3′ REV 5′ CAGACAGGACCAACCGGACT-3′; custom ordered as cDNA oligos from IDT, Coralville, IA, USA) on a Stratagene Mx3005P Instrument (Agilent Technology, Santa Clara, CA, USA). The results were analyzed using the Δ cycles to threshold (ΔCt) method.

### 2.8. Cell Viability Assay

A 3--(4,5--dimethylthiazol--2--yl)--2,5--diphenyltetrazolium bromide (MTT; Sigma--Aldrich) assay was completed to determine cell viability following pharmacologic intervention and therapeutic administration. Cells were plated at 1000 cells/well in a 96-well plate and incubated overnight with or without 100 µM of Buthionine sulfoximine (BSO) (AC309475000; Fisher Scientific). Cells were treated with a serial dilution of cis-Diammineplatinum(II) Dichloride (Cisplatin) (D3371; TCI Chemicals, Portland, OR, USA) for 72 h. After 72 h, plates were incubated with MTT solution for 1.5–2 h. Formazan crystals were dissolved using dimethyl sulfoxide and absorbance was immediately measured at 570 nm using a BioTek Synergy H1 Multimode Reader (Aligent Technology). The half-maximal inhibitory concentration (IC50) was determined using Graphpad Prism Version 10.5.0 (Graphpad Software, La Jolla, CA, USA).

### 2.9. In Silico Validation

RNA-seq count data from cisplatin-sensitive A2780 and cisplatin-resistant A2780cis ovarian cancer cells (GSE98230) was obtained from the Gene Expression Omnibus (accessed 22 May 2026) [19]. Differential expression analysis was performed using DESeq2 (v1.52.0) with default parameters. Genes were considered significantly differentially expressed at FDR < 0.05. Gene Set Enrichment Analysis (GSEA) was performed using the fgsea R package (version 1.38.0) with KEGG Legacy pathway gene sets from MSigDB. Genes were ranked by the product of log2 fold change and −log10(*p*-value).

R scripts for data acquisition, preprocessing, differential expression analysis (DESeq2), and visualization were developed with assistance from Claude (Anthropic, version 3.5 Sonnet, 2026, accessed on 21 May 2026). All analytical outputs and biological interpretations were independently reviewed and verified by the research team.

### 2.10. Statistical Analysis

In the experiments shown, samples within the same treatment group were collected from at least three biologic replicates. Graphs and statistical analyses were generated and completed using Graphpad Prism Version 10.5.0 (Graphpad Software, La Jolla, CA, USA).

Statistically significant differences were determined by unpaired *t*-tests (non-syngeneic samples) or paired *t*-tests (syngeneic samples), as appropriate unless otherwise noted. *p*-values of less than 0.05 were considered significant. Outliers were not removed.

## 3. Results

### 3.1. Chronic Cisplatin Exposure Is Associated with Distinct Metabolic Reprogramming in HGSOC Cells

**Figure 1:**
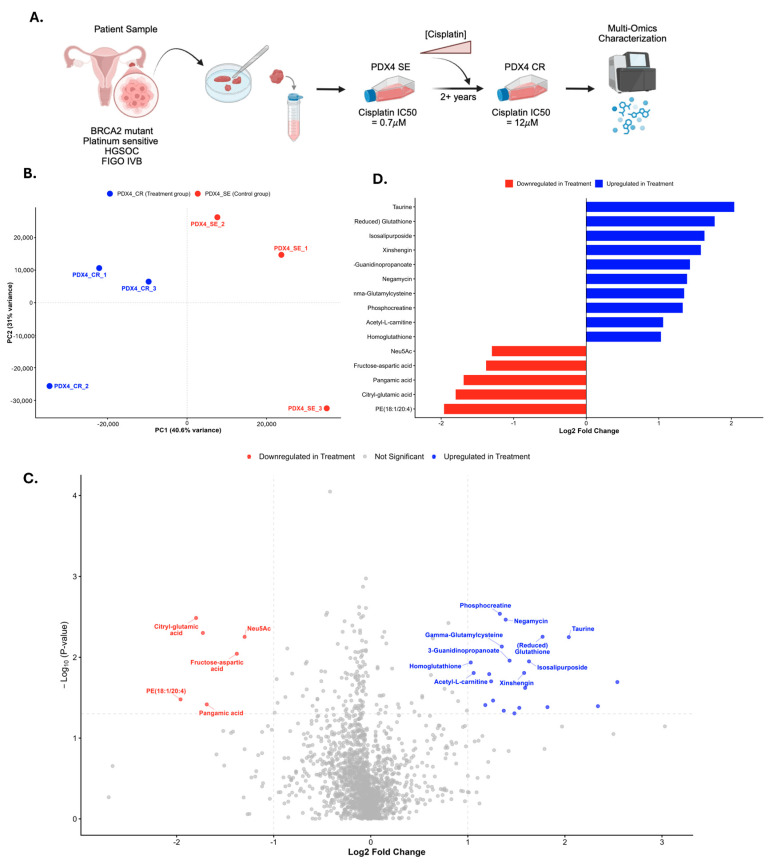
Chronic cisplatin exposure drives metabolic rewiring. (**A**) Schematic of experimental design. Created with BioRender.com. (**B**) PCA of CR and SE samples in biologic triplicate (*n* = 3). (**C**) Volcano plot of differentially expressed metabolites in CR (averaged), compared to SE (averaged) with the top 10 upregulated and top 5 downregulated metabolites labeled. (**D**) Fold change bar chart of the top 10 most up- and downregulated metabolites in CR, compared to SE.

To determine how chronic cisplatin exposure directs metabolic reprogramming associated with the development of chemoresistance, a unique syngeneic pair of chemo-sensitive (SE) and chemoresistant (CR) patient-derived (PDX4) HGSOC cells were utilized. The chemoresistant cells were developed as previously described, through prolonged and increasing exposure to cisplatin in vitro (Figure 1A) [18]. The paired model provides a controlled system to study adaptations that emerge during the pressured development of platinum resistance. To assess these changes, RNA sequencing and untargeted metabolic profiling were performed using Liquid Chromatography–Mass Spectrometry (LC-MS) on both CR and SE cells.

Principal component analysis (PCA) of the metabolomic data obtained revealed distinct separation of SE and CR cell populations along the primary axis (PC1, explaining 40.6% of the variance), indicating that chronic cisplatin exposure is associated with metabolic divergence between the two cell states (SE/CR) (Figure 1B). Although the secondary axis (PC2, explaining 31% of the variance) demonstrated vertical spread among individual replicates, this within-group variability did not obscure the strong overall treatment effect. The distinct clustering seen suggests that resistant cells undergo coordinated metabolic reprogramming rather than isolated alterations in individual metabolites, as demonstrated by the quadrant specific aggregation of the SE samples [quadrant I (top-right) and quadrant IV (bottom-right)] and the CR samples [quadrant II (top-left) and quadrant III (bottom-left)], indicating separate pattern classifications between the two sample cell types. A PCA loadings plot was generated to visualize the contribution of metabolites to each principal component (Figure S2).

To identify the metabolites contributing most significantly to the group separation, top PCA loadings compared with the significant metabolites were identified in the dual-threshold volcano plot. Following data processing and dual thresholding, a total of 21 upregulated metabolites and 6 downregulated metabolites were found to be significantly differentially abundant within the CR cells (Figure 1C). This integrated analysis highlights a marked upregulation of taurine and reduced glutathione, two metabolites associated with oxidative stress buffering, the two most highly upregulated metabolites in CR cells, relative to the SE counterpart (Figure 1D).

This data indicates that chronic cisplatin exposure is associated with metabolic reprogramming in HGSOC cells, characterized by the enrichment of metabolites linked to antioxidant defense. These metabolic adaptations may represent mechanisms by which tumor cells maintain redox balance and survive prolonged exposure to platinum-based therapy.

### 3.2. Chronic Cisplatin Exposure Promotes Glutathione, Taurine, and Amino Acid-Related Pathway Enrichment

Cisplatin is a platinum-based alkylating-like agent that drives disruptions in DNA replication and transcription, ultimately leading to cell cycle arrest and induction of apoptosis (Figure S1) [13]. In addition to DNA damage, cisplatin induces substantial oxidative stress through the generation and accumulation of reactive oxygen species (ROS) and disruption to cellular redox balance. The stress of these toxic effects creates strong selective pressure for survival of tumor cells capable of maintaining antioxidant defense and redox balance during prolonged treatment [20].

**Figure 2:**
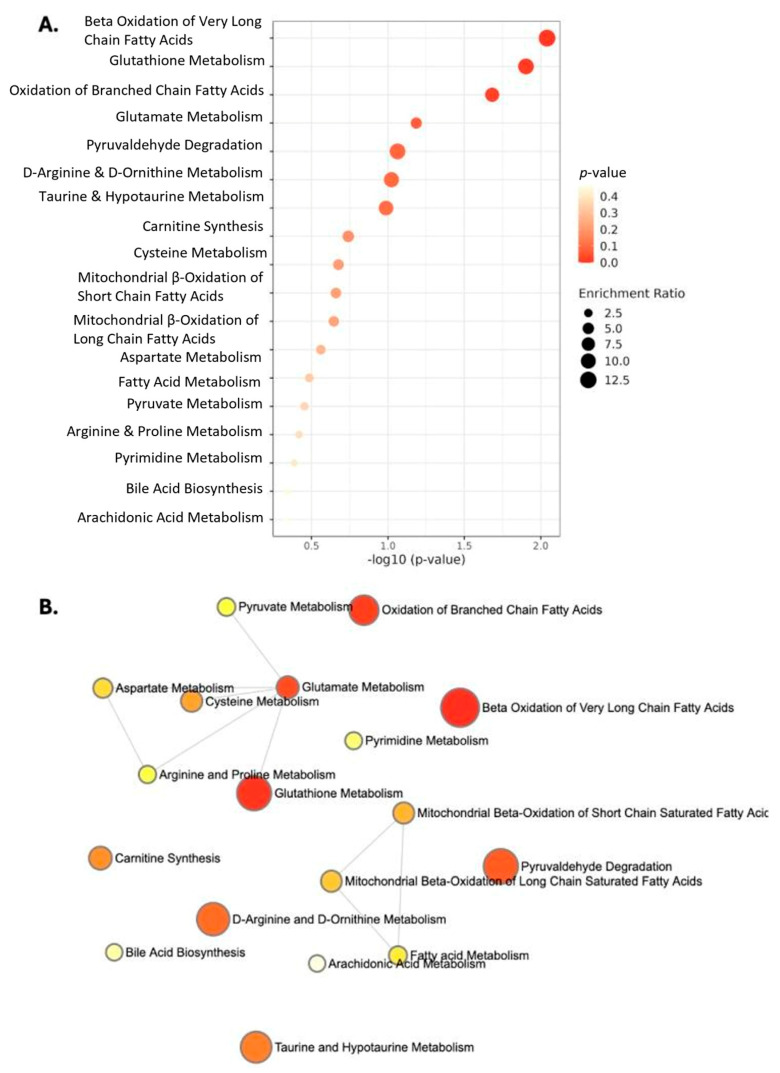
Pathway enrichment analysis of metabolites defines cisplatin-induced metabolic reprogramming. (**A**) Summary plot for overrepresentation analysis using MSEA by MetaboAnalyst 6.0. (**B**) Network analysis visualization from MSEA results. Node color represents enrichment ratio as computed by Hits/Expected, where hits = observed hits; expected = expected hits, and node size reflects pathway impact or enrichment magnitude. Node color represents the *p*-value (lower *p*-values are red/orange, while higher *p*-values are yellow/white). Spatial proximity reflects relative connectivity and does not represent a quantitative distance.

To investigate metabolic pathway enrichment following chronic cisplatin exposure, overrepresentation pathway analysis of the differentially expressed metabolites was completed using Metabolite Set Enrichment Analysis (MSEA) by MetaboAnalyst 6.0. Basic investigation was consistent with the previous individual metabolite identification (Figure 1D), revealing enrichment of pathways related to glutathione metabolism, amino acid metabolism, and taurine metabolism (Figure 2A). Interestingly, and consistent with prior publications observing associations of various metabolites with platinum-resistance, β oxidation of very long chain fatty acids was significantly enriched in the CR cells [21]. Network analysis visualization from MSEA results revealed both direct and indirect associations between glutathione and amino acid metabolism, as evidenced by connection with a line or by distance, suggesting that related metabolic adaptations that provide reducing power and substrates to manage oxidative stress have occurred (Figure 2B).

### 3.3. Cisplatin Resistance Is Associated with Increased Glutathione and Cysteine-Derived Antioxidant Metabolism

To further investigate pathway-level alterations identified by the metabolomic profiling, RNA sequencing of the CR and SE cells was performed to assess metabolic-related gene expression signatures. A total of 7506 differentially expressed genes (DEGs) were identified with 3890 genes downregulated and 3616 genes upregulated, as previously detailed [18].

**Figure 3:**
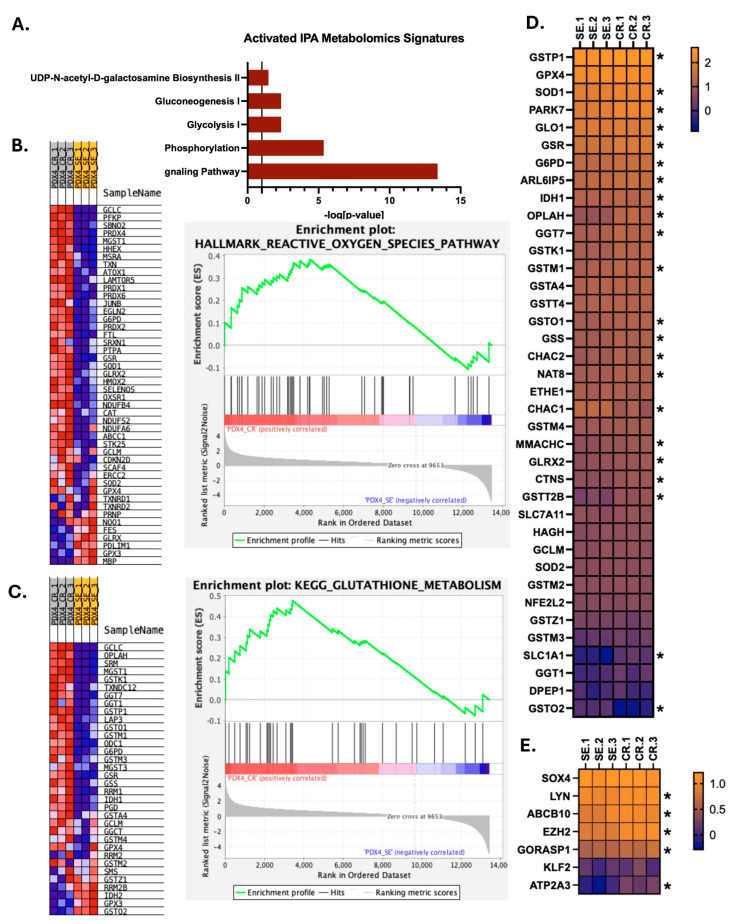
RNA sequencing reveals gene expression changes consistent with metabolic reprogramming toward antioxidant metabolism. (**A**) Ingenuity Pathway Analysis of metabolomics pathways significantly activated in CR cells compared to SE cells. (**B**) GSEA enrichment plots and corresponding blue and pink o’grams indicating significant enrichment of gene sets associated with reactive oxygen species pathways and (**C**) glutathione metabolism in the CR cells. (**D**) Heat map demonstrating differential expression of genes associated with glutathione biosynthesis, metabolism, and recycling [31] as well as (**E**) taurine metabolism [33] in cancer. Blue represents lower gene expression and orange represents higher gene expression levels. Data is shown as log(10) of gene transcripts per million (TPM). Statistical significance was determined using the unpaired *t*-test. *p*-values: *p* ≤ 0.05 (*).

Enhanced stress response, antioxidant, and detoxification mechanisms are key for tumor survival under the toxic conditions of cisplatin-induced cell damage and thus represent areas of interest in this dataset [22]. RNA sequencing of the cells was analyzed using Ingenuity Pathway Analysis (IPA), specifically filtering for metabolomic signatures. Five metabolic signatures were found to be significantly activated in the CR cells, compared to SE, further supporting a cisplatin resistance-associated metabolic shift (Figure 3A).

UDP-N-acetyl-D-galactosamine is a nucleotide sugar utilized by glycotransferases in the biosynthesis of glycoproteins, glycolipids, and mucin-type O-glycan, which enhances protein glycosylation and is associated with poor prognosis in ovarian cancer [23,24]. Gluconeogenesis provides metabolic substrates, intermediates, and NADPH to support other metabolic processes, such as glutathione recycling [25]. Glycolysis provides cells with rapid ATP that feed into pathways such as the pentose phosphate pathway, to produce NADPH and other sources of reducing power [26] to support metabolic shifts required to survive cisplatin treatment. Oxidative phosphorylation is heavily associated with cisplatin resistance, by increasing energy productive and mitochondrial antioxidant capacity to neutralize ROS [27]. Finally, microautophagy signaling facilitates selective degradation of recycling and damaged organelles and oxidized macromolecules to limit ROS accumulation and supply amino acids required for other metabolic processes, such as de novo glutathione synthesis [28].

These pathways represent potential substrate sources for processes involved in intracellular detoxification. As previously explored through the metabolic data, glutathione metabolism is one of the primary defense mechanisms against oxidative and electrophilic stress, as it promotes detoxification of ROS and buffers redox states [29,30]. Thus, further analysis of metabolomic-related signaling was completed using Gene Set Enrichment Analysis (GSEA). Gene sets associated with both ROS and glutathione metabolism were significantly enriched in the CR cells, compared to SE, further suggesting glutathione metabolism as a key antioxidant metabolic process enriched through prolonged cisplatin exposure (Figure 3B,C and Figure S3).

Further analysis of the RNA sequencing was completed using a cancer-specific, reference glutathione metabolism-related gene signature [31], revealing significant increases in several genes, including enzymes involved in glutathione biosynthesis (GCLC and GSS) indicative of enhanced capacity for de novo glutathione production in CR cells (Figure 3C). GCLC RNA expression was validated, and confirmed enrichment observed within the RNA sequencing (Figure S4A). Upregulation of GSR, which regenerates reduced glutathione (GSH) from oxidized glutathione (GSSG), indicates increased recycling capacity to maintain pools of active glutathione. Glutathione utilization enzymes GSTP1 and GSTM1, which operate by conjugating glutathione with electrophilic substrates to protect cells from oxidative stress, suggest enhanced capacity for drug detoxification. Finally, upregulation of cysteine transporter SCL1A1 and enhancement of the γ-glutamyl cycle via upregulation of GGT1 and OPLAH, as well as decreased GGACT (an enzyme involved in degradation and recycling of glutathione), suggests that CR cells have increased glutathione production (de novo and increasing cystine availability), but fail to break down glutathione/convert 5-oxoproline into glutamate to feed back into the cycle (Figure 3C,D). These transcriptional and metabolic changes indicate that the CR cells have increased glutathione production and utilization, while decreasing glutathione breakdown and recycling, in order to mitigate cisplatin-induced oxidative stress.

We previously identified that the CR cells demonstrate an enrichment of metabolic pathways associated with substrate availability, including amino acid metabolism and taurine metabolism (Figure 2A). Amino acids are essential building blocks for antioxidant synthesis and metabolic adaptation consistent with the support that metabolic reprogramming requires. Taurine, a metabolite derived from cysteine metabolism, has been reported to act as an antioxidant by stabilizing mitochondrial function and buffering oxidative stress [32]. Key genes involved in the biosynthesis and metabolism of taurine were called from the RNA sequencing and demonstrate some mild enrichment within the CR cells (Figure 3E).

Several enzymes involved in NADPH generation, a reducing agent essential for regenerating reduced glutathione (GSH), were also increased. G6PD (Glucose-6-Phosphate Dehydrogenase) and PGD (6-Phosphogluconate Dehydrogenase), operating within the oxidative pentose phosphate pathway, as well as ME1 (Malic Enzyme 1) and IDH1 (Isocitrate Dehydrogenase 1), increase levels of available NADPH. Thus, pathways that support the basic needs of increased glutathione metabolism are also increased (Figure S4B).

Collectively, these findings suggest that chronic cisplatin exposure drives coordinated metabolic reprogramming toward cysteine-derived antioxidant defense, enabling tumor cells to increase glutathione-dependent detoxification while generating additional redox-protective metabolites like taurine to support survival under sustained oxidative stress.

### 3.4. Glutathione Metabolism Upregulation Is Functionally Associated with Cisplatin Resistance

To determine if upregulation of glutathione synthesis and metabolism functionally contributes to cisplatin resistance in this model, pharmacologic inhibition of antioxidant buffering was explored. One of the most widely utilized and characterized inhibitors of the glutathione pathway is buthionine sulfoximine (BSO). BSO inhibits γ-glutamylcysteine ligase (GCLC) and depletes intracellular glutathione pools to reduce a cell’s ability to detoxify ROS. It is currently in clinical trials in several disease types, although not yet FDA-approved as a therapeutic [34]. In the PDX4 model, BSO may impair the ability of the cells to buffer cisplatin-induced oxidative stress, thus sensitizing cells to cisplatin administration.

**Figure 4:**
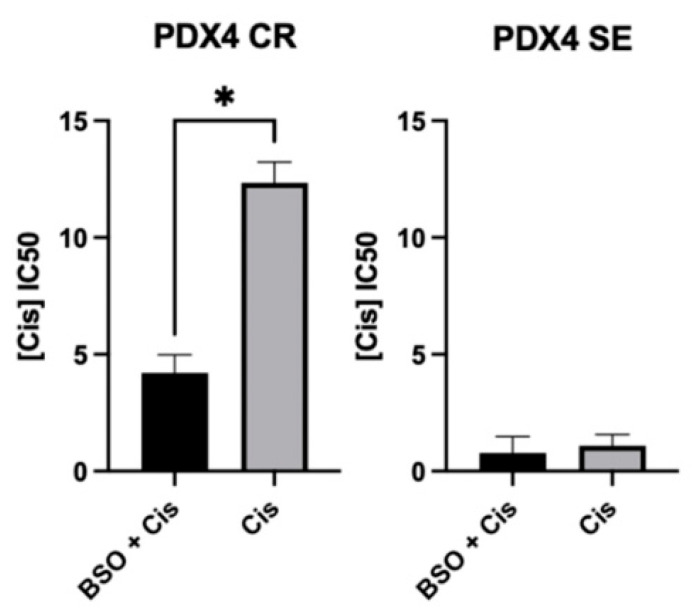
Pharmacologic inhibition of glutathione synthesis produces differential responses in cisplatin-sensitive and -resistant cells. Cisplatin IC50 [µM] of PDX4 CR and SE following 72 h co-treatment of BSO/vehicle and cisplatin. Results are displayed as *n* = 3 and presented as the means ± SD. Statistical significance was determined using the unpaired *t*-test. *p*-values: *p* ≤ 0.05 (*).

To evaluate the contribution of enhanced glutathione metabolism to cisplatin resistance, a cell viability assay was performed. Cells were pre-treated with vehicle or 100µM BSO prior to co-treatment with cisplatin for 72 h. Treatment with BSO in combination with cisplatin significantly sensitized PDX4 CR cells to cisplatin, supporting the hypothesis that the resistant cells rely on adaptive, enhanced glutathione metabolism to buffer cisplatin-induced oxidative stress. Alternately, the SE cells demonstrated no change in cisplatin response with BSO, indicating that these cells do not rely on glutathione-mediated detoxification for survival following cisplatin exposure (Figure 4).

This data suggests that glutathione metabolism may represent an acquired metabolic vulnerability in cisplatin-resistant cells, rather than a universal mediator of platinum response across any cellular state.

### 3.5. Upregulation of Glutathione Synthesis Is a Consistent Feature of Acquired Resistance

While antioxidant buffering has been found to be upregulated and functionally relevant in the PDX4 model, further validation was completed to determine the wider relevance of this shift. RNA sequencing of additional cisplatin-sensitive/-resistant cell lines was mined for genes involved in glutathione synthesis and recycling, including GCLC, GSS, GSR, OPLAH, GGACT, and GPX1. A2780/A2780cis (resistant) was generated through repeated or chronic exposure to platinum-based agents, similar to the generation of the PDX4 model, and provides valuable insight into the generalization of this mechanism4.

**Figure 5:**
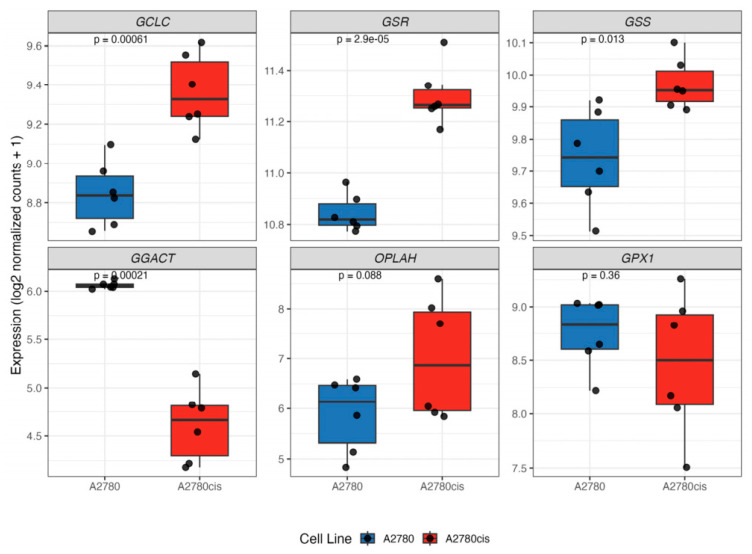
Glutathione synthesis and recycling pathways are enriched in comparative models of acquired cisplatin resistance. Box plots indicating expression (log2 normalized counts +1) of genes involved in glutathione synthesis, recycling, and utilization in A2780/A2780cis cells (GSE98230) [19]. Results are displayed as *n* = 6 per cell type and presented as the means ± SD. Statistical significance was determined using the unpaired *t*-test with *p*-values displayed.

Within the A2780 model of acquired resistance, both GCLC and GSR (enzymes involved in glutathione synthesis and recycling) are highly significantly upregulated in cisplatin-resistant cells, validating the primary findings within PDX4 CR. GSS, an enzyme involved in the second step of glutathione synthesis, was also found to be significantly upregulated (Figure 5). GCLC RNA expression was further validated in an A2780/A2780cis cell line, indicating higher expression of gene in the cisplatin-resistant line (Figure S5). Interestingly, when looking at glutathione recycling, GGACT was found to be downregulated while OPLAH and GPX1 remain unchanged (Figure 5).

These results demonstrate a consistent upregulation of glutathione biosynthesis and recycling pathways in an additional model of acquired cisplatin resistance, supporting and generalizing the observations made in the PDX4 model.

## 4. Discussion

**Figure 6:**
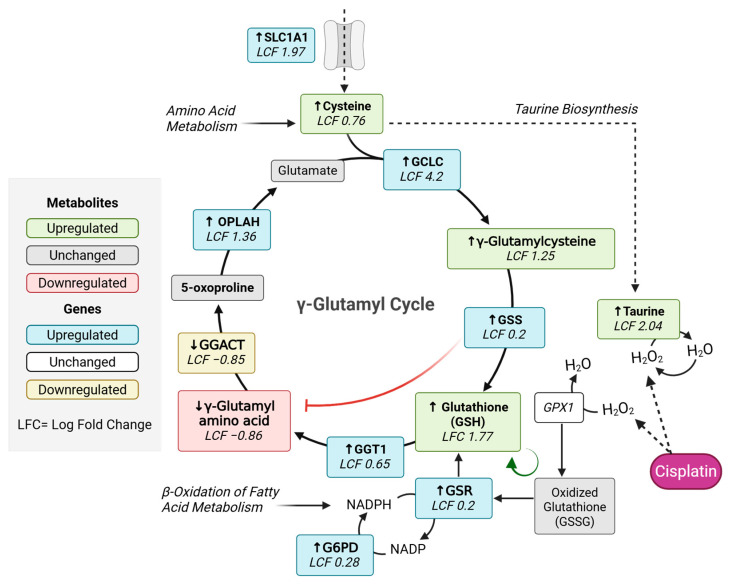
Integrated metabolic adaptation model. Schematic illustrating coordinated metabolic pathways, discussed above, that contribute to sustained redox homeostasis following chronic cisplatin treatment. Cysteine, supplied intracellularly and extracellularly via amino acid metabolism and cystine importers such as SLCA1A, provides a substrate for the γ-glutamyl cycle, supporting glutathione biosynthesis and allowing detoxification of intracellular reactive oxygen species (ROS). NADPH production, derived from both β oxidation of fatty acids and the pentose phosphate pathway, supports glutathione recycling through glutathione reductase activity. Additionally, increased taurine, synthesized from sulfur-containing amino acids like cysteine, also contributes to cellular detoxification by buffering ROS. Created with BioRender.com.

Platinum-based chemotherapy regimens, such as carboplatin or cisplatin, remain the frontline treatment for HGSOC. However, the development of acquired resistance represents a major barrier to consistent clinical response. In the current study, we investigated the metabolic consequences of chronic cisplatin exposure in a syngeneic pair of therapy-sensitive and -resistant patient-derived HGSOC cells using metabolomics and transcriptomic analyses. We demonstrate that chronic cisplatin exposure drives coordinated metabolic rewiring characterized by enrichment of glutathione metabolism, increased activity of NADPH-generating pathways, β oxidation of fatty acid metabolism, and enhanced amino acid metabolism (Figure 6). These adaptations, when taken together, suggest that resistant cells undergo a metabolic shift enhanced to maintain redox homeostasis and mitigate cisplatin-induced oxidative stress.

Strong enrichment of glutathione metabolism within the CR cells was present at both the metabolite level, as well as through pathway enrichment analysis of RNA sequencing data. Both metabolomic and RNA sequencing analyses demonstrated increased glutathione abundance, as well as increased expression of key enzymes involved in glutathione synthesis, recycling, and utilization. Glutathione plays a key role in protecting cells from oxidative damage and stress by detoxifying electrophilic compounds generated through the cellular action of cisplatin. Increased availability of glutathione may enable the resistant cells to mitigate the ROS generated through chronic cisplatin treatment to reduce intracellular toxicity. Further, transcriptional modification of intermediates and enzymes within the γ-glutamyl cycle suggest remodeling of the pathway to favor glutathione turnover and conversion. Notably, taurine metabolism was enriched within the CR cells, indicating a shift toward antioxidant defense. This data is consistent with the concept that increased glutathione levels are associated with cisplatin resistance, suggesting that glutathione metabolism may contribute to sensitivity to oxidative stress and cisplatin response.

Glutathione metabolism, as well as the γ-glutamyl cycle, requires two things: reducing power and amino acids. Evidence from both the metabolomics and the RNA sequencing suggests amino acid metabolism as well as β oxidation of fatty acid metabolism are increased within the CR cells. These pathways all contribute to the availability of cytosolic NADPH, an essential reducing agent which aids in maintaining redox balance and detoxification. Thus, cells can provide the reducing power requirement necessary to drive metabolic reprogramming that promotes robust antioxidant capacity under the chronic stress of cisplatin-induced damage.

RNA sequencing of an additional model of acquired cisplatin resistance, A2780/A2780cis, demonstrated upregulation of genes involved in glutathione synthesis (GCLC, GSR, GSS), downregulation of a key glutathione recycling enzyme (GGACT), and stable expression of GPX1, consistent with the PDX4 model [19]. This data indicates that cisplatin-induced transcriptional reprogramming is associated with disruption of progression through the γ-glutamyl cycle and enhancement of glutathione-mediated detoxification pathways, suggesting that resistant cells preferentially shift metabolism toward maintenance of intracellular redox balance and platinum detoxification. These findings suggest that glutathione metabolic rewiring may be a conserved adaptive mechanism underlying acquired resistance across ovarian cancer models.

The metabolic adaptations observed in the CR cells may create therapeutic vulnerabilities that can be exploited pharmacologically. The strong shift toward glutathione metabolism and NADPH production suggests that the resistant cells may have become dependent on redox-buffering pathways to survive cisplatin-induced stress. Targeting these pathways may sensitize recurrent or resistant tumors to platinum therapy and aid in overcoming resistance. Co-treatment with BSO produced differential effects in SE and CR cells. The CR cells demonstrated a decrease in resistance following BSO/cisplatin administration, indicating the cells may be addicted to this specific mechanism of intracellular detoxification. The SE cells did not demonstrate a change in cisplatin response following BSO administration. The data indicates that glutathione metabolism may not be the dominant survival pathway in the sensitive state but is rather a therapeutically targetable mechanism of acquired resistance. These findings suggest that redox-targeting therapies may be beneficial for patients demonstrating acquired resistance following first-line exposure to platinum-based agents. Given the limited availability of targeted second-line treatment options beyond re-administration of platinum for patients with HGSOC, a combination of platinum with redox-targeting therapies should be considered.

Similar to BSO, several other established and experimental therapies have been evaluated for their role in inhibiting glutathione and related pathways in disease. APR-246 (eprenetapopt), a small molecule drug that acts to reactivate mutant P53, has been shown to react with cysteine residues to deplete glutathione pools and further promote ferroptosis [35,36]. Ethacrynic acid (EA) is a loop diuretic used in the treatment of edema, renal disease, and malignancy-associated ascites management. It is capable of forming a conjugate with glutathione to be a potent inhibitor of glutathione S-transferases and deplete cellular GSH. Clinically, there have been some successes of EA in sensitizing cancer cells to alkylating agents, such as cisplatin [37,38]. Erastin, or similar analogs such as PRLX 93936, are potent ferroptosis inducers used both experimentally and in clinical trials [39,40]. The molecule is a potent inhibitor of the cystine/glutamate antiporter to suppress cystine uptake and contribute to GSH and taurine depletion. This inactivates glutathione peroxidase 4 (GPX4), promotes the accumulation of ROS, and induces ferroptotic cell death. DHEA (Dehydroepiandrosterone) is an inhibitor of G6PHD, which is a source of NADPH in the pentose phosphate pathway [41]. Inhibition of G6PHD reduces production of NADPH and limits NADPH-dependent oxidative stress defenses, such as glutathione metabolism (Table 1). These therapies may be beneficial as co-treatments with cisplatin or other agents, to reduce the cell’s ability to buffer ROS and other forms of oxidative stress (Table 1).

## 5. Conclusions

Overall, this study provides evidence that chronic cisplatin exposure drives coordinated metabolic rewiring in HGSOC cells exposed to chronic cisplatin treatment. This metabolic reprogramming enhances pathways that support antioxidant defense and redox homeostasis. By integrating metabolomic and transcriptomic analyses, we identify glutathione metabolism, amino acid pathways, and NADPH-generating metabolism as central components of the chemoresistance metabolic phenotype. These findings improve our understanding of how tumor cells adapt to prolonged and repeated chemotherapy exposure and suggest that targeting redox metabolic pathways may represent a promising strategy for overcoming platinum resistance in ovarian cancer.

## Figures and Tables

**Table 1:** Pharmacologic strategies targeting glutathione, NADPH, and cysteine metabolism in chemoresistant HGSOC.

Target	Drug Example	Rationale
Glutathione synthesis	BSO Ethacrynic acid	Depletes GSH pool
Glutathione Utilization	APR-246	Deplete glutathione, induce ferroptosis
Cysteine uptake	Erastin	Limits substrate for GSH + taurine
Pentose phosphate pathway	DHEA	Reduces NADPH

## Data Availability

The data is contained within the article and Supplementary Materials.

## Supplementary Materials

The following supporting information can be downloaded at: https://www.mdpi.com/article/10.3390/cancers18121945/s1, Figure S1: Schematic of the mechanism of action of cisplatin. Cisplatin enters the cell via 1) passive diffusion, 2) transporter-mediated uptake, or 3) endocytosis. Once in the cytoplasm, it undergoes aquation, wherein one or both chlorine ions are replaced by water. This activated form of cisplatin localizes to the mitochondria and/or the nucleus, where it forms DNA adducts at specific residues. In parallel, cisplatin induces the generation of reactive oxygen species, further driving DNA damage. Collectively, the effects of cisplatin can be broadly characterized as inducing cellular stress responses that drive adaptive reprogramming and/or cell death. Created with BioRender.com; Figure S2: PCA loadings plot. Top 10 metabolites contributing to PC1 and PC2; Figure S3: GSEA. Plots indicating varying enrichment of pathways related to glutathione and taurine metabolism; Figure S4: Gene Validation and NADPH Generation Pathways. A. Validation of GCLC expression (left) by RT-qPCR (right). B. NADPH-generating enzymes from RNA sequencing. Results are displayed as *n* = 3 and presented as the means ± SD. Statistical significance was determined using the unpaired *t*-test. *p*-values: *p* ≤ 0.05 (*); Figure S5: GCLC Gene Validation. Validation of GCLC RNA expression in A2780/A2780cis cell lines via RT-qPCR. Results are displayed as *n* = 3 and presented as the means ± SD. Statistical significance was determined using the unpaired *t*-test. *p*-values: *p* ≤ 0.05 (*).
