# Supplementary material for: Multi-Omics Analysis Reveals Chronic Cisplatin Exposure Is Associated with Metabolic Rewiring Toward Glutathione Metabolism to Support Redox Adaptation in High-Grade Serous Ovarian Cancer
Source: Cancers (Basel). 2026 Jun 15;18(12):1945. doi: 10.3390/cancers18121945 (PMC13296594; doi:10.3390/cancers18121945)
Supplement: Supplementary file 1 [file cancers-18-01945-s001.zip › cancers-4332864-supplementary.pdf]

Multi-Omics Analysis Reveals Chronic Cisplatin Exposure Is Associated with Metabolic Rewiring Toward Glutathione Metabolism to Support Redox Adaptation in High-Grade Serous Ovarian Cancer

Ashlyn Conant [1], Kayla Sanchez [2], Shreya Patil [3], Ethan Nyein [4], Tise Suzuki [1,†], Gary Yu [1], Marlon Maus [5], Salvador Soriano [1], Christian Hurtz [3] and Juli J. Unternaehrer [1,4,*]

[1] Department of Basic Sciences, School of Medicine, Loma Linda University, 11085 Campus Street Mortensen Hall 219, Loma Linda, CA 92354, USA; abartlett@students.llu.edu (A.C.); suzukit@southern.edu (T.S.); gyu@llu.edu (G.Y.); ssoriano@llu.edu (S.S.)

[2] The Taub Institute for Research on Alzheimer's Disease and the Aging Brain, Vagelos College of Physicians and Surgeons, Columbia University, New York, NY 10032, USA; ks4561@cumc.columbia.edu

[3] Division of Cancer Sciences, Department of Basic Sciences, Loma Linda University, Loma Linda, CA 92354, USA; shreyapatil@llu.edu (S.P.); churtz@llu.edu (C.H.)

[4] Department of Gynecology and Obstetrics, Loma Linda University, Loma Linda, CA 92354, USA; enyein@llu.edu

[5] School of Public Health, The University of California, Berkeley, CA 94720, USA; maus1@berkeley.edu

* Correspondence: junternaehrer@llu.edu; Tel.: +1-(909) 558-7691; Fax: +1-(909) 558-4887

† Current Address: Department of Biology and Allied Health, Southern Adventist University, Collegedale, TN 37315, USA.

Supplementary Material

The following supplementary material is available. S1: Schematic of the mechanism of action of cisplatin. S2: PCA loadings plot. S3: GSEA. S4: Gene Validation and NADPH Generation Pathways. S5: GCLC Gene Validation. S5: GCLC Gene Validation.

Supplemental Data

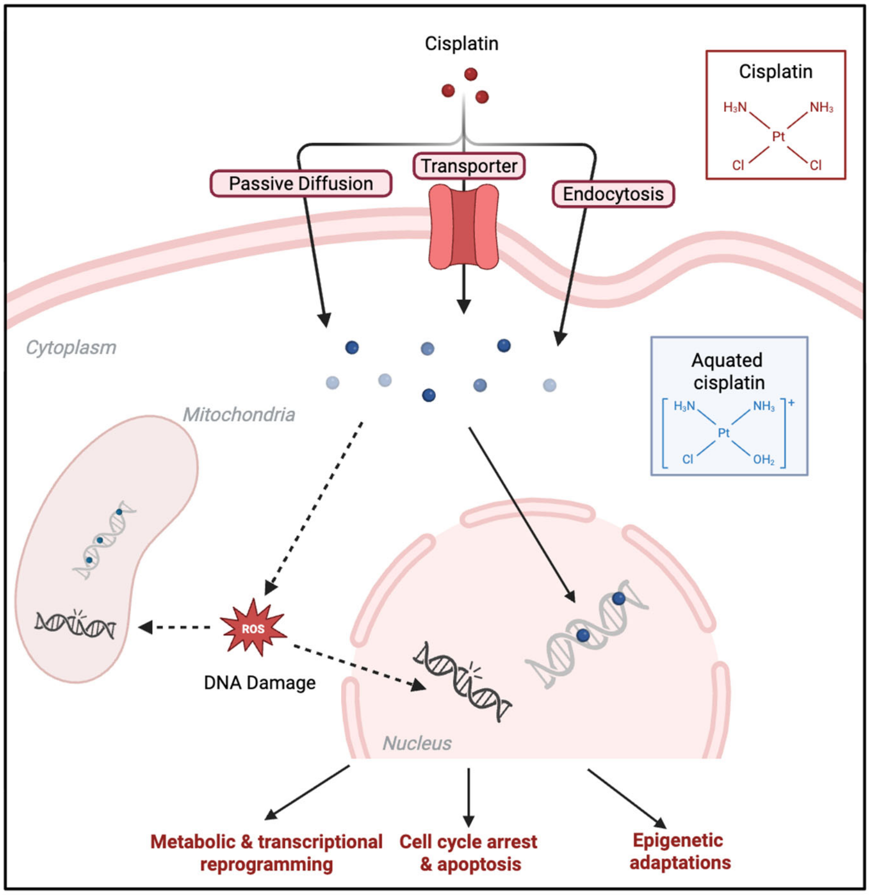

Supplementary Figure S1: Schematic of the mechanism of action of cisplatin. Cisplatin enters the cell via 1) passive diffusion, 2) transporter-mediated uptake, or 3) endocytosis. Once in the cytoplasm, it undergoes aquation, wherein one or both chlorine ions are replaced by water. This activated form of cisplatin localizes to the mitochondria and/or the nucleus, where it forms DNA adducts at specific residues. In parallel, cisplatin induces the generation of reactive oxygen species, further driving DNA damage. Collectively, the effects of cisplatin can be broadly characterized as inducing cellular stress responses that drive adaptive reprogramming and/or cell death. Created with BioRender.com.

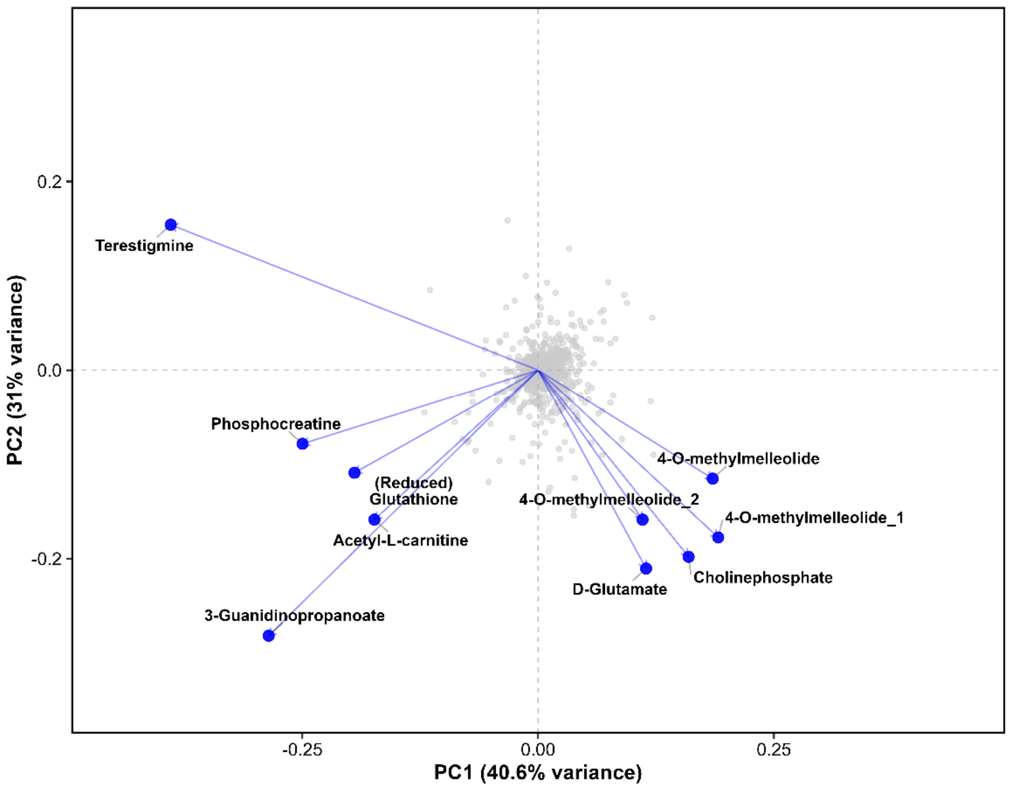

Supplementary Figure S2: PCA loadings plot. Top 10 metabolites contributing to PC1 and PC2.

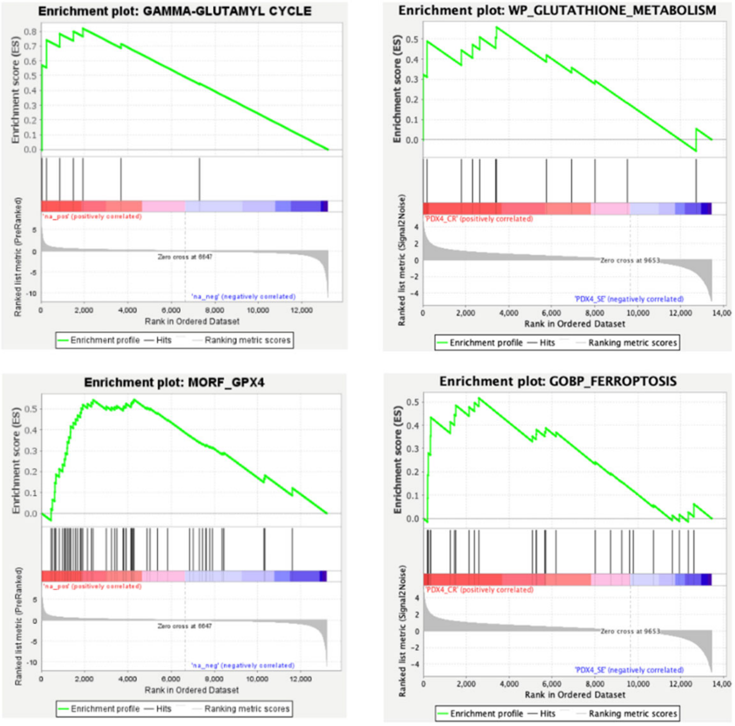

Supplementary Figure S3: GSEA. Plots indicating varying enrichment of pathways related to glutathione and taurine metabolism.

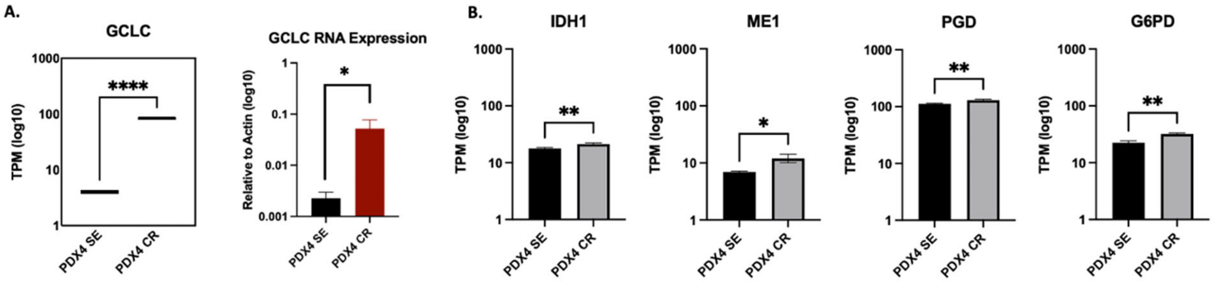

Supplementary Figure S4: Gene Validation and NADPH Generation Pathways. A. Validation of GCLC expression (left) by RT-qPCR (right). **B**. NADPH-generating enzymes from RNA sequencing. Results are displayed as $n = 3$ and presented as the means ± SD. Statistical significance was determined using the unpaired t-test. *p*-values: $p \leq 0.05$ (*).

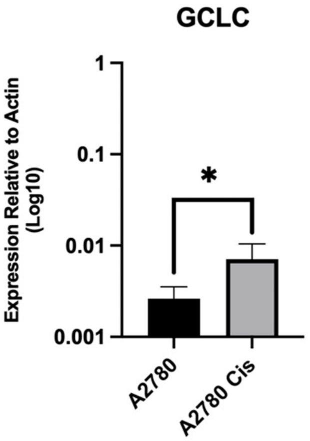

Supplementary Figure S5: GCLC Gene Validation. Validation of GCLC RNA expression in A2780/A2780cis cell lines via RT-qPCR. Results are displayed as $n = 3$ and presented as the means ± SD. Statistical significance was determined using the unpaired t-test. *p*-values: $p \leq 0.05$ (*).
